# Occurrence of *Fusarium* Mycotoxins in Cereal Crops and Processed Products (*Ogi*) from Nigeria

**DOI:** 10.3390/toxins8110342

**Published:** 2016-11-18

**Authors:** Cynthia Adaku Chilaka, Marthe De Boevre, Olusegun Oladimeji Atanda, Sarah De Saeger

**Affiliations:** 1 Laboratory of Food Analysis, Department of Bioanalysis, Faculty of Pharmaceutical Sciences, Ghent University, Ottergemsesteenweg 460, 9000 Ghent, Belgium; marthe.deboevre@ugent.be (M.D.B.); sarah.desaeger@ugent.be (S.D.S.); 2 Department of Food Science and Technology, College of Applied Food Science and Tourism, Michael Okpara University of Agriculture, Umuahia-Ikot Ekpene Road, Umudike, PMB 7267 Umuahia, Abia State, Nigeria; 3 Department of Biological Sciences, McPherson University, KM 96 Lagos-Ibadan Expressway, 110117 Seriki Sotayo, Ogun State, Nigeria; olusegunatanda@yahoo.co.uk

**Keywords:** *Fusarium* mycotoxins, occurrence, cereal, *ogi*, LC-MS/MS, Nigeria

## Abstract

In Nigeria, maize, sorghum, and millet are very important cash crops. They are consumed on a daily basis in different processed forms in diverse cultural backgrounds. These crops are prone to fungi infestation, and subsequently may be contaminated with mycotoxins. A total of 363 samples comprising of maize (136), sorghum (110), millet (87), and *ogi* (30) were collected from randomly selected markets in four agro-ecological zones in Nigeria. Samples were assessed for *Fusarium* mycotoxins contamination using a multi-mycotoxin liquid chromatography-tandem mass spectrometry (LC-MS/MS) method. Subsequently, some selected samples were analysed for the occurrence of hidden fumonisins. Overall, 64% of the samples were contaminated with at least one toxin, at the rate of 77%, 44%, 59%, and 97% for maize, sorghum, millet, and *ogi*, respectively. Fumonisins were the most dominant, especially in maize and *ogi*, occurring at the rate of 65% and 93% with mean values of 935 and 1128 μg/kg, respectively. The prevalence of diacetoxyscirpenol was observed in maize (13%), sorghum (18%), and millet (29%), irrespective of the agro-ecological zone. Other mycotoxins detected were deoxynivalenol, zearalenone, and their metabolites, nivalenol, fusarenon-X, HT-2 toxin, and hidden fumonisins. About 43% of the samples were contaminated with more than one toxin. This study suggests that consumption of cereals and cereal-based products, *ogi* particularly by infants may be a source of exposure to *Fusarium* mycotoxins.

## 1. Introduction

Mycotoxins are secondary metabolites produced by a wide diversity of toxigenic fungi, which often contaminate crops worldwide [1]. These fungi are ubiquitous in nature, and may contaminate crops in the field or during storage, thus producing mycotoxins under favourable environmental conditions. *Fusarium* fungi are of high significance because of their ability to cause several devastating plant diseases, and being responsible for economic losses and trade barriers, while having potential in producing a wide range of mycotoxins. *Fusarium* mycotoxins have been linked to several health related problems in animals and humans ranging from acute (such as anorexia and diarrhoea) to chronic disease conditions (such as cancer and immunosuppression) [2,3]. For instance, fumonisins when ingested are carcinogenic, neurotoxic, and hepatotoxic and may possibly lead to death [1,2,4]. Efforts to understand the production and behaviour of mycotoxins, and to protect consumers from mycotoxicoses have led to extensive investigation of these toxins across the globe as well as establishment of regulatory maximum limits by the developed countries. Although it is estimated that several *Fusarium* mycotoxins do exist in nature, those mostly studied are the fumonisins (FB), trichothecenes (TH), and zearalenone (ZEN). This is due to the high toxic effects they exert on humans and animals, and their frequent occurrence in agricultural products especially cereals and cereal-based food products. 

Cereals such as maize (*Zea mays*), sorghum (*Sorghum bicolor*), and millet (*Pennisetum glaucum*) serve as major staple crops consumed especially by the middle and low income earners in Nigeria. These crops are often processed into different food forms including processing of traditional weaning meal in the region. *Ogi* (also known as akamu) is a fermented cereal-based product used as a major traditional weaning food for infants, food for the convalescent and the elderly as well as consumed by different age groups especially as breakfast meal in Nigeria. It is produced by submerge fermentation of cereal grains (maize, sorghum, or millet) for two to three days followed by wet milling and sieving through a mesh. The fermentation process of *ogi* is usually initiated by chance inoculation under uncontrolled environmental conditions thereby resulting in variable quality of the final product. Studies have reported the prevalence of *Fusarium* mycotoxins, particularly TH, ZEN, and FB, in cereal crops and cereal-based products globally [5,6,7,8]. In most cases, these mycotoxins may co-exist in food and food products which often results to a synergistic, additive or antagonistic toxic effect on the host [9]. The mixed effects of mycotoxins have been revealed by the study of Kouadio et al. [10] on the effects of combinations of ZEN, fumonisin B_1_ (FB_1_), and deoxynivalenol (DON) on the human intestinal cell line (Caco-2). FB_1_ in combination with ZEN showed lesser effect on the reduction of cell viability when compared to the combined effect of FB_1_ with other mycotoxins because of the antagonistic effect of FB_1_ on ZEN [10]. Similarly, Speijer and Speijer [9] observed the antagonistic effect of DON on T-2 in the inhibition of human lymphocytes proliferation. It is noteworthy to mention that ternary combination of type B TH (fusarenon-X (FUS-X), nivalenol (NIV), and DON) exhibited an antagonistic interaction on the intestinal epithelial cells which is possibly linked to a lower toxicity of FUS-X in the mixture [11]. There exist a potential relationship in the reduction of FUS-X toxicity and the competition between DON and NIV at the substrate binding sites of the de-acetylase thus leading to a reduced deacetylation of FUS-X [11]. Cases of synergistic interaction exhibited by combination of mycotoxins such as ZEN, DON, and FB_1_ have also been reported [10,12]. Harvey et al. [13] and Kubena et al. [14] demonstrated the synergistic and additive effects resulting to growth depression in pigs and broiler chicks, respectively because of co-occurrence of mixed mycotoxins (DON and FB_1_). A synergistic interaction between several combinations of type B TH on epithelial cell toxicity has also been recorded [11,15]. Recently, issues of possible co-existence of these *Fusarium* mycotoxins and their modified forms have become of great concern. Modification of mycotoxins may be triggered by food processing, or matrix related, or through conjugation by either plant, fungi or animal [16,17]. These modified mycotoxins often escape routine analysis leading to underestimation of actual mycotoxin levels in products and may possibly hydrolysed into the parent toxins during digestion [18]. Several studies on the occurrence of *Fusarium* mycotoxins in cereals and cereal-based products have reported the natural occurrence and co-occurrence of modified mycotoxins such as DON-3-glucoside (DON-3G), ZEN-4-glucoside (ZEN-14G), and α- and β-zearalenol-4-glucoside (α- and β-ZEL-4G) [5,19]. The possible underestimation of FB concentration in cereals and cereal-based products as a result of presences of hidden FB has been demonstrated [20,21,22]. Hidden FB cannot be directly analysed as they have to be released from the matrix into extractable form (hydrolysed FB) by sample treatment often by alkaline hydrolysis [23,24].

The increasing rate of climate change, which is characterised by significant increase or decrease in temperature and/or alteration of rainfall during planting season in sub-Saharan Africa (especially Nigeria), may have predisposed this region to *Fusarium* mycotoxins contamination. Evidence of possible occurrence of *Fusarium* mycotoxins in Nigeria is revealed by the frequent incidence of major mycotoxin producing *Fusarium* species such as *F. verticillioides*, *F. graminearum*, *F. poae*, *F. proliferatum*, and *F. sporotrichioides* in Nigerian food commodities [25,26]. In spite of this obvious evidence, limited study has been undertaken to ascertain the possible occurrence of *Fusarium* mycotoxins and their modified forms in Nigeria food products. This has resulted to the lack of regulatory maximum levels governing the control of *Fusarium* mycotoxins in Nigeria. Sub-Saharan African countries including Nigeria solely depend on maximum levels set by the European Union and the Codex Alimentarius Commission on control of *Fusarium* mycotoxins without considering the feeding habits and other socio-economic dynamics faced by this region. The main objective of the present paper is to investigate the occurrence of *Fusarium* mycotoxins and their modified forms including fumonisin B_1_, B_2_, and B_3_; hidden FB; DON; 3-acetyl-DON (3ADON); 15-acetyl-DON (15ADON); DON-3G; ZEN; α-zearalenol (α-ZEL); β-zearalenol (β-ZEL); ZEN-14G; NIV; FUS-X; T-2 toxin (T-2); HT-2 toxin (HT-2); diacetoxyscirpenol (DAS); and neosolaniol (NEO) in Nigerian cereals—maize, sorghum, millet, and the processed products (*ogi*).

## 2. Results and Discussion

### 2.1. Fusarium Mycotoxins Contamination in Cereals (Maize, Sorghum, and Millet) and Processed Products (Ogi) from Nigeria

A total of 363 samples comprising maize (*n* = 136), sorghum (*n* = 110), millet (*n* = 87), and *ogi* (*n* = 30) were evaluated for the occurrence of *Fusarium* mycotoxins and modified forms including FB_1_, FB_2_, FB_3_, hidden FB, DON, 3ADON, 15ADON, DON-3G, ZEN, α-ZEL and β-ZEL, ZEN-14G, NIV, FUS-X, T-2, HT-2, DAS, and NEO. These samples were collected from randomly selected markets from four agro-ecological zones in Nigeria between September 2015 and October 2015. Out of the 18 *Fusarium* mycotoxins analysed in the samples, 15 toxins were present in at least one of the samples. Data on the incidence and occurrence level of individual *Fusarium* mycotoxins in the cereals (maize, sorghum, and millet) and processed products (*ogi*) are illustrated in Table 1 and Table 2. Over 40% prevalence rate of the mycotoxins was recorded in all sample types, with the individual rate of 77%, 44%, 59%, and 97% for maize, sorghum, millet, and *ogi*, respectively.

Maize, sorghum, millet, and *ogi* contained 13, 13, 10, and 14 *Fusarium* secondary metabolites, respectively, of which only four (FB_1_, FB_2_, DON, and ZEN) are regulated by the European Union (EU). Fumonisins were the most dominant mycotoxins occurring at high level and incidence rate in all the food types especially in maize and *ogi* samples. The sum of fumonisins (FB_1_ + FB_2_ + FB_3_ (FB)) were in the ranges of 32–8508 μg/kg (65%), 45–180 μg/kg (8%), 74–22,064 μg/kg (14%), and 125–3557 μg/kg (93%) in maize, sorghum, millet, and *ogi*, respectively. Except for sorghum, most of the maize and millet samples in this study exceeded the maximum regulatory limit set for the sum of FB_1_ and FB_2_ (1000 μg/kg) by the European Union (EU) [27] suggesting the high exposure of the population to this toxin. A similar high FB incidence rate has been reported in several studies from sub-Saharan Africa [28,29,30,31,32,33,34] at concentrations ranging up to 53,863 μg/kg [30]. High incidence of FB, especially in maize may be explained by the susceptibility of the maize crop to FB producing fungi (*F. verticillioides* and *F. proliferatum*) [35]. Sorghum and millet had a much lower incidence rate, however an extreme concentration of FB was recorded in one of the millet samples (22,064 μg/kg). Lower concentrations and incidence rate in sorghum and millet from Ethiopia have previously been reported [36]. However, the reversed trend was observed by Ayalew et al. [37], who recorded higher levels of FB (range: 1370–2117 μg/kg) in sorghum samples. Of the FB, FB_1_ occurred at a more frequent rate than FB_2_ and FB_3_. Although, we observed that some of the millet (*n* = 4) and maize (*n* = 16) samples were contaminated with only FB_2_. Such trend has previously been reported in cereals suggesting the possible contamination of *Aspergillus niger,* which is a principal producer of FB_2_ [38]. The study of Ezekiel et al. [39] on sorghum grain confirms the possible occurrence of only FB_2_ in cereals from Nigeria. The incidence and levels of FB as observed in *ogi* is of concern. This present study reveals for the first time the occurrence of *Fusarium* mycotoxins in *ogi* from Nigerian market. The maximum concentration and percentage incidence of FB_1_, FB_2_, and FB_3_ detected in *ogi* samples were 1903 μg/kg (93%), 1,283 μg/kg (87%), and 371 μg/kg (77%), respectively (Table 1). About 83% of the *ogi* samples exceeded the EU maximum limit of 200 μg/kg for processed maize-based foods for infants and young children [27]. Interestingly, out of the 30 *ogi* samples analysed, the only two FB negative samples were of sorghum-base. This confirms the previous study that sorghum is less prone to fungal infestation than maize [39]. Although there are no available data on the occurrence of *Fusarium* mycotoxins in *ogi*, studies from the same country reported the occurrence of FB in two fermented traditional cereal-based beverages (kunu-zaki and pito) [39].

The next group of dominating mycotoxins were the TH. They have been associated with the temperate regions, however studies emerging from sub-Saharan Africa have revealed the possible occurrence of these toxins in the tropics. Type B TH DON, 15ADON, DON-3G, and NIV were detected in our samples. DON was present in 16%, 3%, 13%, and 13% of maize, sorghum, millet, and *ogi* samples at a maximum level of 225 μg/kg, 119 μg/kg, 583 μg/kg, and 74 μg/kg, respectively. Interestingly no sample, irrespective of the food type, exceeded the EU maximum limit (1750 μg/kg, maize; 1250 μg/kg, other cereals; and 200 μg/kg, cereal-based infant foods) for DON [27]. Incidence of DON, as observed in this study, was similar to that reported in previous studies on Nigerian cereals [26,40], but much less than that reported in maize by Adetunji et al. [32] and Ediage et al. [34]. The same trend was reported in millet, sorghum, and cereal-based food samples from Burkina Faso [29]. Studies have shown the occurrence of acetylated DON forms and DON-3G in maize and its products [31,41]. Maize samples in our study were negative for 3ADON, 15ADON, and DON-3G. This is in agreement with a previous study on maize from Burkina Faso [29]. Sorghum, millet, and *ogi* were contaminated with 15ADON, and were negative of 3ADON. The production of acetylated derivatives (15ADON and 3ADON) by *F. graminearum* have been reported and the potential of the isolates to produce 15ADON or 3ADON as the major isomer is dependent on the geographic origin [42,43]. Although the information on the regional relationship between *F. graminearum* and the production of 15ADON or 3ADON is still lacking in Africa, Li et al. [44] and Mirocha et al. [42] reported the predominant of 3ADON in New Zealand, Austrialia, and China while 15ADON chemotype is predominant in North America. The glucoside of DON (DON-3G) was observed to contaminate sorghum and *ogi* samples in the present study. A comparable result on DON-3G in sorghum and millet from Ethiopia have also been reported [36].

Samples of maize (*n* = 3) and *ogi* (*n* = 2) were contaminated with NIV at concentration ranges of 163–271 μg/kg and 136–160 μg/kg, respectively. Occurrence of NIV in Nigerian maize has previously been reported, although at a higher incidence rate (54%) [32]. While similar result in cereal-based products as shown in this study was reported by Castillo et al*.* [45]. Contrary to the result reported on the occurrence of NIV in sorghum and millet by Chala et al. [36], sorghum and millet were negative for NIV in the present study. The trend observed in this study with NIV was also seen with FUS-X contamination. NEO was not detected in any of the samples analysed.

With regards to type A TH, DAS and HT-2 were present in all the sample types except for the cereal-based products (*ogi*) which was negative for DAS, while T-2 was negative in all the sample types. DAS was detected in maize, sorghum, and millet at a rate of 13%, 18%, and 29%, respectively (Table 1). The concentrations of DAS in the cereals ranged between 2 μg/kg and 25 μg/kg. The occurrence of DAS in the samples is probably associated with the occurrence of major DAS-producing fungi in this region [25,26]. DAS and HT-2 are synthesised by a wide range of *Fusarium* species, and they are alleged to be among the most toxic TH occurring in different food products. Several studies have reported the occurrence of DAS in cereals and cereal-based products [46,47]. Despite its association with the temperate weather, previous studies revealed the occurrence of DAS in the tropical regions. Adejumo et al. [26] and Adetunji et al. [32] recorded the occurrence of DAS in Nigerian maize at maximum concentrations of 51 μg/kg (9%) and 30 μg/kg (19%), respectively . Besides maize, DAS has been found to contaminate sorghum and millet from Ethiopia with maximum concentrations of 64.2 μg/kg (mean value, 11.9 μg/kg), and 1.43 μg/kg (mean value, 1.43 μg/kg), respectively [36]. A total of 1%, 8%, 5%, and 3% of maize, sorghum, millet, and *ogi*, respectively, were positive of HT-2 (Table 1). Beside the low incidence rate, none of the cereals or *ogi* samples exceeded the EU recommendation level of 100 μg/kg and 15 μg/kg for cereal and infant foods, respectively [48].

Other mycotoxins detected in the study include ZEN, α-ZEL, β-ZEL, and ZEN-14G. Recent studies have shown the prevalence of ZEN in food products from sub-Saharan Africa [36,49], however in the present study, ZEN was rarely detected. ZEN was detected in maize, sorghum, and millet at 1%, 1%, and 14%, respectively (Table 1) with the concentrations in all the sample types being less than the EU maximum limit of ZEN, except for millet with 8 samples (9%) exceeding 100 μg/kg [27]. Further, only one sample (3%) of *ogi* was positive for ZEN with the value exceeding the maximum limit of 20 μg/kg set by EU for processed cereal-based foods for infants and young children [50]. About 1% and 2% of maize were contaminated with α-ZEL and β-ZEL, respectively. Although there exist only limited studies on the occurrence of these metabolites in food products from sub-Saharan Africa, available data show their possible occurrence in Nigerian maize [32,49]. The present study is in agreement with the result of Adetunji et al. [32]. The maximum levels for β-ZEL in sorghum, millet, and *ogi* were 21 μg/kg, 39 μg/kg, and 20 μg/kg, respectively. Millet samples were negative of α-ZEL while sorghum and *ogi* had 3% and 7% incidence rate with maximum levels of 33 μg/kg and 22 μg/kg, respectively (Table 1). Chala et al. [36] reported a higher incidence rate of α-ZEL and β-ZEL in sorghum and millet compared to the current study, however, the levels reported by these authors were lower. With regards to ZEN-14G, all food type samples analysed showed positive samples with maximum concentrations of 24 μg/kg (maize), 22 μg/kg (sorghum), 34 μg/kg (millet), and 31 μg/kg (*ogi*). Occurrence of this modified form of ZEN in cereals and *ogi* in the current study is supported by a study which detected a wide range of modified forms of ZEN in cereal-based food products [18,19]. Although, there are no recommendation nor regulation limit of ZEN-14G in cereals and cereal-based products because of the non availability of toxicological data, the occurrence of ZEN-14G as observed in the present study is presumed to add additional toxic effect to the host. De Boevre et al. [51] reported the possible hydrolysis of ZEN-14G into its parent form (ZEN) in the digestive tract of mammals suggesting an additional toxicity.

### 2.2. Fumonisins and Hidden Fumonisins Contamination in Cereals (Maize, Sorghum and Millet) and Processed Products (Ogi) from Nigeria

To determine the occurrence of hidden fumonisins, samples were selected from each food type based on the FB result obtained from the multi-mycotoxin analysis (Table 1). Five FB positive and five FB negative samples of each food type (maize, sorghum, and millet) were selected for analysis. Note that eight positive samples and two negative samples of *ogi* were used for the analysis because only two samples of *ogi* were negative. Each of the samples were analysed simultaneously for FB (FB_1_, FB_2_, and FB_3_) as well as total fumonisins after hydrolysis as described in Section 4. Calculation of hidden FB concentration was based on the difference between the concentration of FB and the concentration of total FB after hydrolysis [52]. The maximum FB and total FB concentration in the selected samples of maize, sorghum, millet, and *ogi* samples were 3514 and 4568 μg/kg, 180 and 502 μg/kg, 840 and 3059 μg/kg, and 1496 and 1795 μg/kg, respectively. After hydrolysis, we observed an increment ranging from 1.3 to 5.2 times higher levels of total FB in maize samples. The same trend was observed in sorghum, millet, and *ogi* samples. Hidden FB have been alleged to occur in processed products especially nixtamalised and thermally processed foods [52,53]. However, recent studies have revealed the occurrence of these toxins in unprocessed food products especially in raw maize samples [20,54] which suggest the possible transformation of FB to bound derivatives by natural phenomena due to plant metabolism [24]. The presence of hidden FB as observed in the current study may pose an additional health risk to consumers especially to the consumers of *ogi* analysed in this study. FB has been alleged to cause a range of toxic health effect on humans and animals especially in sub-Saharan Africa where cases of very high levels of FB have been recorded. Cases of human oesophageal cancer in South Africa and other parts of the World have been linked to the consumption of food contaminated with FB. Since it is obvious that hidden FB may cause additional toxic effect on the host as observed when low FB contaminated feed was fed to animals [55], the occurrence of hidden FB in cereals and cereal-based products should no longer be neglected especially in Nigeria where these products serve as major staple food.

### 2.3. Distribution of Fusarium Mycotoxins in Major Cereals across the Different Agro-Ecological Zones of Nigeria

Mycotoxin occurrence and distribution is influenced by different factors including crop species, climatic, and environmental conditions of a given region. The mean and maximum concentrations of individual mycotoxins in the different food types and AEZ are shown in Table 3. Fumonisins contaminations were observed in all the cereal types irrespective of the AEZ. Sudan Savanna (SS) and Northern Guinea Savanna (NGS) zones had the highest incidence rate of FB_1_ in maize with a highest FB_1_ concentration of 2443 μg/kg and 8222 μg/kg, respectively, when compared to Southern Guinea Savanna (SGS) and Derived Savanna (DS). A similar trend with FB_1_ contamination was also seen when the sum of FB (FB_1_, FB_2_, and FB_3_) was considered. This is also similar with the result obtained from the sorghum samples, with the SS zone registering the highest FB_1_ followed by NGS and SGS. This observation could be linked to the high mycotoxins production potentials of *Fusarium* fungi in warmer climates [56] and the significant change in climatic conditions in this region characterised by increase in rainfall and longer raining seasons [57]. Among the other mycotoxins detected, DAS was the next most common metabolite contaminating all the food types across the AEZ, although at lower concentrations. While there are no existing regulatory limits set for DAS in food products, DAS has been implicated in a wide range of toxic effects in animals as well as human, ranging from acute to chronic. It has been linked to a human fatal disease (alimentary toxic aleukia), exhibiting several symptoms such as inflammation of the skin, vomiting, and damage to hematopoietic tissues [1,3]. DON also occurred in the respective cereal types across the AEZ, except for the sorghum samples from the SGS zone (Table 3). The highest incidence rate of DON was observed in maize samples from DS zone characterized by a lower temperature and higher average annual rainfall of 25–35 °C and 1300–1500 mm, respectively when compared to the other zones. In millet samples, the NGS zone registered the highest incidence rate of DON. Comparing the incidence of ZEN across the different food types and AEZ, samples from SS zones were negative of ZEN regardless of the cereal type. This result could be related to the prevailing local weather conditions of this region which is between 30 and 40 °C, which is above the optimum temperature of 25 °C for the production of ZEN [58].

**Figure 1:**
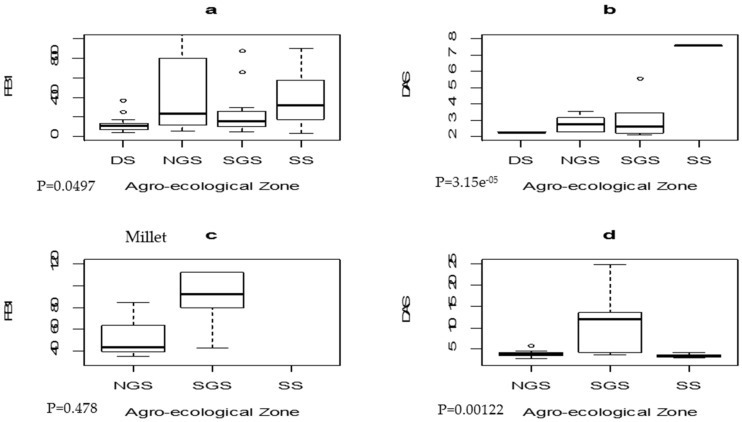
Differences in *Fusarium* mycotoxins in maize ((**a**) fumonisin B_1_; and (**b**) diacetoxyscirpenol); and millet ((**c**) fumonisin B_1_; and (**d**) diacetoxyscirpenol) across agro-ecological zones.

In general, it is postulated that *Fusarium* fungi and subsequent mycotoxins occurrence is higher in colder regions. We observed the trend in the incidence of *Fusarium* toxins in this study with approximately colder region having multiple mycotoxins (Table 3). Further analysis to assess the significant difference in the distribution of *Fusarium* mycotoxins across the AEZ was done using the Kruskal Wallis test (Figure 1). With respect to maize, there was a significant difference in FB_1_ and DAS contamination across the AEZ, whereas in millet samples, DAS contamination was significantly different across the different zones. The less difference observed across the AEZ in the current study may be attributed to the quality of the sampled food products because of the premium placed on high quality (visual observation) food products which arises through sorting and cleaning in order to add more value to the products, thus increasing the market value of the food product.

### 2.4. Co-Occurrence of Fusarium Mycotoxins in Cereals and Processed Products (Ogi) from Nigeria

**Figure 2:**
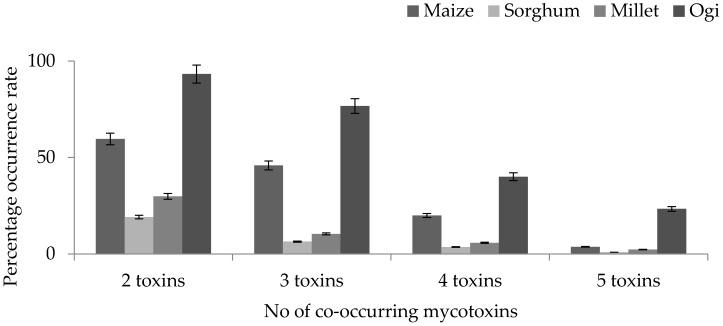
Percentage of co-occurrence of *Fusarium* mycotoxins in maize, sorghum, millet, and *ogi* from Nigerian markets.

Occurrence of multiple mycotoxins in food, especially cereals and cereal-based products has been an issue of great concern because of the synergistic and/or additive effects caused by the interaction of these toxins in humans and animals. In this study, we observed that 60%, 19%, 30%, and 93% of maize, sorghum, millet, and *ogi*, respectively, were contaminated with at least two mycotoxins (Figure 2). In maize samples, FB co-existed with DAS at 13%, being the highest level observed, followed by co-occurrence between FB and DON (11%). DAS and DON-3G co-occurred in 16% of sorghum samples, succeeded by DON-3G and HT2, while DAS and ZEN dominated in millet samples followed by DAS and DON. Previous studies have shown the frequent co-contamination of *Fusarium* toxins in cereal products in sub-Saharan Africa [32,34,36], which is linked to co-occurrence of several species of *Fusarium* fungi in crops or the potential ability of one *Fusarium* spp. to produce more than one mycotoxins [59]. However, the rates observed in sorghum and millet in the present study were in contrast with the data reported on co-occurrence of mycotoxins in sorghum (94%) and millet (85%) from South Korean retail markets [60], and may be attributed to the different sampling regions considered for these studies.

Although only limited studies exist on the co-occurrence of DON, ZEN, and their modified forms in sub-Saharan Africa, available data proves the potential of DON co-occurring with its modified forms in cereals from this region [34]. This was not the case in this study. The disparity may be attributed to the differences in sampling protocol. Ediage et al. [34] analysed household samples, while samples from the markets were analysed in the present study. Cereals purchased from the market often are of better quality compared to those from the household or at farmers disposal. Ironically, this is because high-graded cereal grains are often placed for sale by the farmers because of a better market bargain, while they retain the poor quality cereal grains since there are no provisional channels for destruction or diversion of these products.

Interestingly, the processed cereal-based products (*ogi*) analysed in this study had the highest co-occurrence rate compared to the cereals. This observation could be attributed to many factors (such as poor sanitation during processing). *Ogi* is fermented under uncontrolled conditions, and as such could be contaminated with any form of organism residing in the environment. Another possible avenue for contamination is the quality of the raw cereal used in the production of *ogi*. Although reduction of mycotoxin concentrations have been reported during processing of *ogi* [61], it is important to mention that reduction of mycotoxins during food processing is dependent on the initial concentrations of the raw produce and as such good quality cereal grains should be used for production of *ogi*. Up to 23% of *ogi* samples were contaminated with at least five mycotoxins, with the highest co-occurrence existing between FB and DON-3G, and subsequently between FB and DON. DON and DON-3G co-occurred in four of the samples while ZEN, ZEN-14G, α-ZEL, and β-ZEL co-occurred in one sample. This result is affirmed by the previous study on co-occurrence of these metabolites in cereal-based products [5]. However, the rates and concentration levels in their study were higher than the levels observed in this study.

## 3. Conclusions

The present study showed the occurrence of *Fusarium* toxins in cereals and cereal-based fermented products (*ogi*) in Nigeria. Fumonisins were the most dominating *Fusarium* mycotoxins with some of the samples exceeding the FB regulatory limits set by the EU. Although the levels of other mycotoxins detected in the samples were low, the co-occurrence of these mycotoxins presents a health risk due to the synergistic and/or additive effect, considering the fact that these food products are consumed almost on daily basis. In addition, it is worrisome that the traditional weaning food fed to a large population of infant and growing children in this region contains high levels of FB with a cocktail of other mycotoxins. This phenomenon shows that infants are affected with different variation of toxic effects. Reducing mycotoxins in *ogi* is important, and mainly determined by the quality of the raw cereal before fermentation. Therefore, there is need to educate the small-scale producers on the risk of mycotoxins and possible ways to reduce or avoid contamination. Furthermore, it is worth mentioning that though all cereals were contaminated with varying degrees of toxins, the incidence rate was higher in maize, suggesting sorghum and millet as a possible alternative. These results suggest the need for a national coordinated food safety action plan.

## 4. Materials and Methods

### 4.1. Sampling

**Figure 3:**
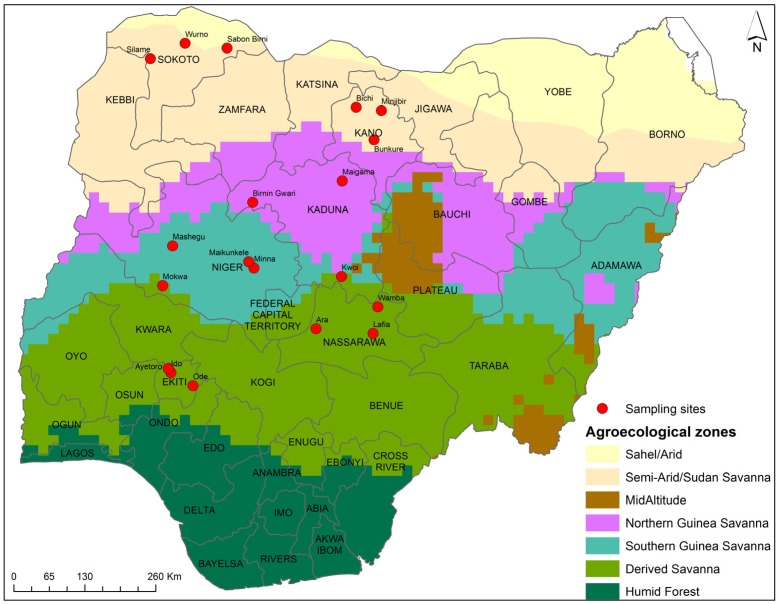
Map of Nigeria showing the sampling sites of maize, sorghum, millet, and *ogi*.

Nigeria has seven agro-ecological zones (AEZ) (Figure 3) based on the climatic and environmental conditions, out of which four AEZ were selected for sampling which include the Derived Savanna (DS), Southern Guinea Savanna (SGS), Northern Guinea Savanna (NGS), and Sudan Savanna (SS). The sampling sites were considered on the production areas of the crops and products. One state from each AEZ was covered for each crop and product. Maize samples were collected from four AEZ while millet and sorghum were sampled from SGS, NGS, and SS; and *ogi* samples were collected from DS. Table 4 shows the detailed sampling sites.

The geographical location, temperature, and rainfall pattern of DS, SGS, NGS, and SS are documented by Udoh et al. and Atehnkeng et al. [62,63]. Briefly, the DS is characterized by an annual rainfall distribution and temperature ranging between 1300 mm and 1500 mm, and 25 °C to 35 °C, respectively. The average annual rainfall of SGS is between 1000 mm and 1300 mm with a temperature range of 26 to 38 °C. The NGS has rainfall distribution averaging between 900 mm and 1000 mm annually, and temperatures vary from 28 to 40 °C, while SS is characterised by an annual rainfall distribution between 650 mm and 1000 mm annually, and temperatures varying from 30 to 40 °C.

A total of 363 samples comprising of maize (*Zea mays*) (*n* = 136), sorghum (*Sorghum bicolor*) (*n* = 110), millet (*Pennisetum glaucum*) (*n* = 87), and *ogi* (*n* = 30) were collected from randomly selected markets between September 2015 and October 2015. All cereal samples were sorted and cleaned and had no visible mould before going to the market. Sampling was carried out as described by Adetunji et al. and European Commission Regulation with some modifications [32,64]. Briefly, the whole content of a traditional bag of 50 kg of maize, sorghum, and millet was considered as a lot. An aggregate sample size of 1 kg was composed of 5 incremental samples. Each incremental portion was about 200 g, and one was taken from different positions in the bag. In the case of *ogi*, 300 g of sample was collected from the top, middle, and bottom portions of the jute sack of 10 kg *ogi*, thoroughly mixed together, air dried, and packed in a plastic container. Each sample of the different food products (maize, sorghum, millet, and *ogi*) was thoroughly homogenised, and a representative portion of 200 g was taken, labelled, and sealed in zip lock bags, and transported to the Laboratory of Food Analysis, Ghent University, Belgium (Ottergemsesteenweg 460, Ghent, Belgium) for further analysis. Prior to analysis, maize, sorghum, millet, and *ogi* samples were milled to a sieve size of 0.5–1 mm using an IKA M20 universal mill (Sigma-Aldrich, Bornem, Belgium) and stored at −20 °C.

### 4.2. Chemicals and Reagents

Methanol (MeOH, LC-MS grade), glacial acetic acid (LC-MS grade), and analytical grade acetonitrile were purchased from Biosolve B.V. (Valkenswaard, The Netherlands). Analytical grade acetic acid, ammonium acetate and sodium chloride were obtained from Merck (Darmstadt, Germany). Analytical grade of n-hexane and methanol, Whatman^®^ glass microfiber filters (GFA, 47 mm diameter) were purchased from VWR International (Zaventem, Belgium). Ultrafree^®^-MC centrifugal filter devices (0.22 μm) were obtained from Millipore (Bredford, MA, USA). C18 solid phase extraction (SPE) columns and MultiSep^®^226 AflaZon+ multifunctional columns were purchased from Alltech (Lokeren, Belgium) and Romer Labs (Gernsheim, Germany), respectively. Water was purified using a Milli-Q Gradient System (Millipore; Brussels, Belgium). All other chemicals and reagents used were of analytical grade.

The analytical mycotoxin standards including FB_1_, FB_2_, DON, 3ADON, 15ADON, deepoxy-deoxynivalenol (DOM), FUS-X, NIV, HT-2, NEO, ZEN, zearalanone (ZAN), α-ZEL, β-ZEL were purchased from Sigma-Aldrich (Bornem, Belgium). FB_3_ was obtained from Promec Unit (Tygerberg, South Africa). DAS, DON-3G and T-2 were purchased from Biopure Referenzsubstanze (Tulln, Austria) while ZEN-14G was synthesized via an in-house validated method according to Zill et al. [65]. Stock solutions of FB_1_, FB_2_, FB_3_, DON, 3-ADON, 15-ADON, HT-2, T-2, ZEN, ZAN, α-ZEL, β-ZEL, FUS-X, NIV, NEO, and DAS were prepared in MeOH at a concentration of 1 mg/mL. DOM (50 μg/mL), DON-3G (50.2 μg/mL) and ZEN-14G (100 μg/mL) were obtained as solution in acetonitrile. All stock solutions were stored for 1 year or until the expiration date at −18 °C. The working standard solutions were made by diluting the stock standard solutions in methanol, and were stored at −18 °C for 3 months. From the individual stock and working standard solutions, a standard mixture was prepared in methanol at the following concentrations: FB_1_, FB_2_, and DON (40 ng/μL), FB_3_ (25 ng/μL), 3-ADON (5 ng/μL), 15-ADON (2.5 ng/μL), HT-2, T-2, ZEN, α-ZEL, β-ZEL, ZEN-14G, DON-3G, and NEO (10 ng/μL), FUS-X and NIV (20 ng/μL), and DAS (0.5 ng/μL). The mixture was stored at −18 °C, and renewed every 3 months.

### 4.3. Sample Extraction

Sample preparation for 17 *Fusarium* mycotoxins was carried out as described by Monbaliu et al. [66] for multi-mycotoxin analysis. Briefly, 5 g of sample was spiked with internal standards (ZAN and DOM at a concentration of 250 and 150 μg/kg, respectively). DOM was used as internal standard for DON, DON-3G, 3ADON, and 15ADON while ZAN was used for the other mycotoxins. Spiked sample was kept in the dark for 15 min and extracted with acetonitrile/water/acetic acid (79/20/1, *v*/*v*/*v*). The supernatant was passed through a preconditioned C18-SPE column, and the extract was defatted. In order to recover the 17 *Fusarium* mycotoxins, two clean-up pathways were followed. Firstly, 12.5 mL of the defatted extract was added to 27.5 mL of acetonitrile/acetic acid (99/1, *v*/*v*), and passed through a MultiSep^®^226 AflaZon+ multifunctional column. In the second pathway, 10 mL of defatted extract was filtered using a glass microfilter. Two milliliters of the filtered extract were combined with the Multisep226 eluate and evaporated to dryness. The residue was then redissolved in 150 µL of the mobile phase (water/methanol/acetic acid (94/5/1, *v*/*v*/*v*) + 5 mM ammonium acetate and water/methanol/acetic acid (2/97/1, *v*/*v*/*v*) + 5 mM ammonium acetate in the ratio of 3/2, *v*/*v*). The redisolved mixture was then filtered using the Ultrafree^®^ PVDF centrifuge filters and analysed by LC-MS/MS.

### 4.4. Preparation of the Hydrolysed FB_1_, FB_2_, and FB_3_ Standard

The hydrolysed FB standards were prepared as described by Dall Asta et al. [24]. Briefly, 90 μL standard solution of FB_1_, FB_2_, and FB_3_ (50 μg/mL of each) was prepared in methanol, transferred to a 10 mL sovirel tube, and evaporated to dryness under a gentle stream of nitrogen. The residue was redissolved in 2 M NaOH (1 mL), and the reaction was incubated overnight at room temperature. After hydrolysis, the mixture was extracted by liquid-liquid partition using acetonitrile (1 mL). The extraction was repeated two more times using 1 mL fresh acetonitrile. The organic phases were pooled together, evaporated under a nitrogen stream and redissolved in 1 mL of methanol. The reaction yield was checked by LC-MS, by monitoring the conversion of FB to hydrolysed FB and the absence of side-products, and it was found to be higher than 99%. Calibration curves were prepared by proper dilution of the standard solution, assuming the total conversion of the native compounds to the hydrolysed forms.

### 4.5. Sample Preparation for Hydrolysed Fumonisins

Ten samples from each matrix were selected for analysis of hydrolysed fumonisin; five samples comprising of high FB contaminated samples, while the second five samples were comprised of negative samples. Sample preparation was carried out as described by Dall Asta et al. [24]. Two and half grams of each sample was weighed, and was hydrolysed at room temperature using 50 mL of 2 M NaOH, homogenised with the Ultraturrax for 3 min, and then stirred using a magnetic stirrer for 60 min. Subsequently, 50 mL of acetonitrile was added, and homogenised for 3 min, and 20 mL of the upper organic phase was transferred into a centrifuge tube, and centrifuged for 15 min at 3500 rpm. Then, 4 mL was evaporated, and the residue was redissolved in 1 mL of water/methanol (30/70, *v*/*v*), and analysed by LC-MS/MS.

### 4.6. Liquid Chromatography-Tandem Mass Spectrometry

A Waters Acquity UPLC system coupled to a Micromass Quattro Micro triple-quadrupole mass spectrometer (Waters, Milford, MA, USA) was used for the detection and quantification of mycotoxins in the samples. The data acquisition and processing utilities included the use of the MassLynx™ version 4.1 and QuanLynx^®^ version 4.1 software (Micromass, Manchester, UK). The column used was a Symmetry C18 (150 mm × 2.1 mm i.d. 5 μm) column with a guard column (10 mm × 2.1 mm i.d.) of the same material (Waters, Zellik, Belgium), and was kept at room temperature. The injection volume was 20 μL. Mobile phase consisting of water/methanol/acetic acid (94/5/1, *v*/*v*/*v*) and 5 mM ammonium acetate (mobile phase A), and methanol/water/acetic acid (97/2/1, *v*/*v*/*v*) and 5 mM ammonium acetate (mobile phase B) were used at a flow rate of 0.3 mL/min with a gradient elution program. The gradient started at 95% mobile phase A with a linear decrease to 35% in 7 min. Mobile phase A decreased to 25% at 4 min, and an isocratic period of 100%, mobile phase B started at 11 min for 2 min. Initial column conditions were reached at 23 min using a linear decrease of mobile phase B, and the column was reconditioned for 5 min prior to the following injection. The mass spectrometer was operated using selected reaction monitoring (SRM) channels in positive electrospray ionization (ESI+) mode. More information and on the transition of the different mycotoxins are described by De Boevre et al. [41] and Monbaliu et al. [67]. The capillary voltage was 3.2 kV, and nitrogen was used as the desolvation gas. Source and desolvation temperatures were set at 150 and 350 °C, respectively.

### 4.7. Method Validation

For each matrix, validation parameters including apparent recovery, limit of detection (LOD), limit of quantification (LOQ) and measurement uncertainty were evaluated. Five blank samples of each matrix were spiked in triplicate with the different mycotoxins (FB_1_, FB_2_ and FB_3_, ZEN, α-ZEL, β-ZEL, ZEN-14G, DON, 3ADON, 15ADON, DON-3G, NIV, FUS-X, T-2, HT-2, DAS, NEO) on three different days at concentration levels shown on Table S1. ZAN and DOM were added as internal standards (IS). Matrix-matched calibration (MMC) plots were constructed by applying the least-squares method, and by plotting the relative peak area (peak area of toxin/peak area of IS) against the spiked concentration level of the sample. The linearity was evaluated graphically using a scatter plot, and the linear regression model was tested using a lack-of-fit test. The apparent recovery for each of the mycotoxins was evaluated by dividing the observed value from the MMC curves by the spiked level. The obtained results ranged between 75% and 110%, which is in conformity with the range set in legislation [64].

A precision study with regards to repeatability (intraday precision) and reproducibility (interday precision) within laboratory was performed using five concentration levels, and was calculated using relative standard deviation (RSD). The results are presented in Table S1. LOD and LOQ for individual mycotoxins were obtained from the signal-to-noise (S/N) ratio which have been defined and set as 3 and 10, respectively, by the International Union of Pure and Applied Chemistry (IUPAC). The LOQs of individual mycotoxins in maize, sorghum, and millet are shown in Table S2.

### 4.8. Statistical Analysis

Data were processed and calculated using Microsoft office Excel 2007 (Redmond, WA, USA) and the statistical package R version 3.0.3 (The R Foundation for Statistical Computing, Vienna, Austria). The Kruskal Wallis statistical test (an alternative to one-way analysis of variance (ANOVA) under nonparametric test) was used to assess the significance of the differences between the determined mycotoxin concentrations and the different AEZ.

## Figures and Tables

**Table 1:** Mean and maximum concentration (μg/kg) of *Fusarium* mycotoxins found in cereals and cereal-based products (*ogi*) from Nigeria.

Mycotoxin ^1^	Maize (*n* = 136)			Sorghum (*n* = 110)			Millet (*n* = 87)			*Ogi* (*n* = 30)		
	% + ve Samples ^2^	Mean ^3^	Max ^4^	% + ve Samples	Mean	Max	% + ve Samples	Mean	Max	% + ve Samples	Mean	Max
FB_1_	65	541	8222	8	64	78	9	2333	18,172	93	590	1903
FB_2_	54	376	2885	2	48	55	13	609	3892	87	472	1283
FB_3_	43	117	445	2	38	46	0	na	na	77	121	371
∑FB	65	935	8508	8	83	180	14	2113	22,064	93	1128	3557
DON	16	99	225	3	100	119	13	151	543	13	61	74
15 ADON	0	na ^5^	na	2	39	44	1	11	11	3	60	60
DON-3G	0	na	na	23	24	63	0	na	na	17	30	44
ZEN	1	65	65	1	38	38	14	419	1399	3	39	39
ZEN-14G	9	21	24	3	19	22	6	23	34	3	31	31
α-ZEL	1	20	20	3	33	33	0	na	na	7	20	22
β-ZEL	2	20	21	1	21	21	1	39	39	10	19	20
HT-2	1	20	20	8	20	31	5	36	36	3	13	13
NIV	2	206	271	0	na	na	0	na	na	7	148	160
FUS-X	1	154	154	0	na	na	0	na	na	7	133	137
DAS	13	3	8	18	5	16	29	5	25	0	na	na

^1^ FB_1_, B_2_, and B_3_ = fumonisin B_1_, B_2_, and B_3_; ∑FB = sum of FB_1_, B_2_, and B_3_; DON = deoxynivalenol; 15ADON = 15-acetyl-deoxynivalenol; DON-3G = deoxynivalenol-3-glucoside; ZEN = zearalenone; α-ZEL = α-zearalenol; β-ZEL = β-zearalenol; ZEN-14G = zearalenone-14-glucoside; NIV = nivalenol; FUS-X = fusarenon-X; HT-2 = HT-2 toxin; DAS = diacetoxyscirpenol, ^2^ % + ve Samples = percentage positive samples, ^3^ Mean = mean concentration, ^4^ Max = maximum concentration, ^5^ na = not applicable.

**Table 2:** Contamination levels of fumonisins, total fumonisins, and hidden fumonisin in selected samples.

Food Type	FB (μg/kg)			Total FB (μg/kg)			Hidden FB (μg/kg)		
	Median	Mean	Maximum	Median	Mean	Maximum	Median	Mean	Maximum
Maize (*n* = 10)	358	835	3514	543	1636	4568	144	801	2923
Sorghum (*n* = 10)	41	61	180	95	182	502	50	120	323
Millet (*n* = 10)	118	277	840	302	776	3059	179	499	2254
*Ogi* (*n* = 10)	247	531	1496	391	672	1795	117	141	313

Hidden FB concentration = the difference between the concentration of FB and the concentration of total FB after hydrolysis.

**Table 3:** *Fusarium* mycotoxins occurrence in cereals (maize, sorghum, and millet) across the different agro ecological zones of Nigeria.

Mycotoxin ^1^	Maize (μg/kg)								Sorghum (μg/kg)						Millet (μg/kg)					
	DS (*n* = 30)		SGS (*n* = 36)		NGS (*n* = 40)		SS (*n* = 30)		SGS (*n* = 30)		NGS (*n* = 40)		SS (*n* = 40)		SGS (*n* = 30)		NGS (*n* = 30)		SS (*n* = 27)	
	Mean ^2^ (% + ve) ^3^	Max ^4^	Mean (% + ve)	Max	Mean (% + ve)	Max	Mean (% + ve)	Max	Mean (% + ve)	Max	Mean (% + ve)	Max	Mean (% + ve)	Max	Mean (% + ve)	Max	Mean (% + ve)	Max	Mean (% + ve)	Max
FB_1_	117 (67)	366	249 (33)	876	928 (83)	8222	505 (77)	2443	70 (7)	71	59 (10)	76	67 (8)	78	3700 (17)	18,172	54 (10)	84	na	na
FB_2_	289 (57)	1011	350 (28)	677	508 (65)	2885	295 (70)	1107	na	na	41 (3)	41	55 (3)	55	417 (37)	3892	56 (13)	103	44 (7)	47
FB_3_	114 (47)	353	147 (25)	445	126 (53)	441	91 (50)	213	na	na	31 (3)	31	46 (3)	46	na	na	na	na	na	na
DON	78 (27)	147	99 (17)	180	98 (10)	151	140 (13)	225	119 (3)	119	91 (5)	92	na	na	140 (7)	200	171 (20)	543	118 (11)	118
15 ADON	na ^5^	na	na	na	na	na	na	na	34 (3)	34	44 (3)	44	na	na	na	na	na	na	11 (4)	11
DON-3G	na	na	na	na	na	na	na	na	12 (27)	16	30 (40)	63	22 (3)	22	na	na	na	na	na	na
ZEN	na	na	na	na	65 (3)	65	na	na	38 (3)	38	na	na	na	na	481 (33)	1399	109 (7)	198	na	na
ZEN-14G	20 (23)	24	21 (6)	22	23 (8)	23	na	na	20 (3)	20	19 (5)	22	na	na	29 (7)	34	19(7)	20	23 (4)	23
α-ZEL	na	na	20 (3)	20	na	na	na	na	na	na	33 (8)	33	na	na	na	na	na	na	na	na
β-ZEL	20 (3)	20	na	na	21 (3)	21	na	na	21 (3)	21	na	na	na	na	na	na	39	39	na	na
HT-2	20 (3)	20	na	na	na	na	na	na	19 (17)	19	24 (8)	31	11 (3)	11	35 (7)	35	36 (7)	36	na	na
NIV	228 (7)	271	na	na	na	na	163 (3)	163	na	na	na	na	na	na	na	na	na	na	na	na
FUS-X	154 (3)	154	na	na	na	na	na	na	na	na	na	na	na	na	na	na	na	na	na	na
DAS	2 (10)	2	3 (17)	6	3 (18)	4	8 (7)	8	5 (27)	13	4 (13)	5	5 (18)	16	12 (17)	25	4 (37)	6	3 (33)	4

Abbreviation: DS = Derived Savanna; SGS = Southern Guinea Savanna; NGS = Northern Guinea Savanna; SS = Sudan Savanna. ^1^ FB_1_, B_2_, and B_3_ = fumonisin B_1_, B_2_, and B_3_; DON = deoxynivalenol; 15ADON = 15-acetyl-deoxynivalenol; DON-3G = deoxynivalenol-3-glucoside; ZEN = zearalenone; α-ZEL = α-zearalenol; β-ZEL = β-zearalenol; ZEN-14G = zearalenone-14-glucoside; NIV = nivalenol; FUS-X = fusarenon-X; HT-2 = HT-2 toxin; DAS = diacetoxyscirpenol; ^2^ Mean = mean concentration, ^3^ % + ve = percentage positive samples, ^4^ Max = maximum concentration, ^5^ na = not applicable.

**Table 4:** Sampling sites of maize, sorghum, millet, and *ogi* from different agro-ecological zones of Nigeria.

Product Type	AEZ	State	No. of Markets	No. of Samples
Maize	SS	Kano	6	30
	NGS	Kaduna	6	36
	SGS	Niger	8	40
	DS	Nasarawa	6	30
				Total number = 136
Sorghum	SS	Kano	6	30
	NGS	Kaduna	2	40
	SGS	Niger	8	40
				Total number = 110
Millet	SS	Sokoto	6	30
	NGS	Kaduna	6	30
	SGS	Niger	8	27
				Total number = 87
*Ogi*	DS	Ekiti	5	30
				Total number = 30

Abbreviation: AEZ = agro-ecological zones, SS = Sudan Savanna, NGS = Northern Guinea Savanna, SGS = Southern Guinea Savanna, DS = Derived Savanna.

## Supplementary Materials

The following are available online at www.mdpi.com/2072-6651/8/11/342/s1, Table S1: Intraday repeatability (RSDr, %) and interday reproducibility (RSDR, %) (within laboratory) of the individual mycotoxins for maize, sorghum and millet. Table S2: Limits of detection (LOD, µg/kg) and limits of quantification (LOQ, µg/kg) of the individual mycotoxins for maize, sorghum, and millet.
