# Supplementary material for: Occurrence of Fusarium Mycotoxins in Cereal Crops and Processed Products (Ogi) from Nigeria
Source: Toxins (Basel). 2016 Nov 18;8(11):342. doi: 10.3390/toxins8110342 (PMC5127138; doi:10.3390/toxins8110342)
Supplement: Supplementary file 1 [file toxins-08-00342-s001.pdf]

Supplementary Materials: Occurrence of *Fusarium* Mycotoxins in Cereal Crops and Processed Products (*Ogi*) from Nigeria

Cynthia Adaku Chilaka, Marthe De Boevre, Olusegun Oladimeji Atanda and Sarah De Saeger

Table S1. Intraday repeatability (RSDr) and interday reproducibility (RSDR) of the individual mycotoxins for maize, sorghum, and millet.

Mycotoxin	Calibration Range (μg/kg)	RSDr (%)			RSDR (%)		
		Maize	Sorghum	Millet	Maize	Sorghum	Millet
Fumonisin B_1	200–800	3	5	5	8	7	8
Fumonisin B_2	200–800	6	8	6	8	9	9
Fumonisin B_3	25–100	16	23	12	26	26	39
Deoxynivalenol	200–800	11	14	7	18	20	15
3-acetyl-deoxynivalenol	25–100	13	22	9	17	15	11
15-acetyl-deoxynivalenol	12.5–50	11	16	4	16	19	6
Deoxynivalenol-3-glucoside	5–20	11	6	6	9	7	9
Zearalenone	50–200	7	6	6	11	9	11
α-Zearalenol	50–200	18	18	18	22	22	22
β-Zearalenol	50–200	10	9	10	11	13	11
Zearalenone-14-glucoside	50–200	18	24	16	22	22	15
Nivalenol	100–400	22	24	14	24	16	16
Fusarenon-X	100–400	10	8	9	22	14	14
T-2 toxin	50–200	9	8	13	19	10	10
HT-2 toxin	50–200	7	9	9	7	10	8
Diacetoxyscirpenol	2.5–10	10	15	12	18	17	14
Neosolaniol	50–200	12	12	12	24	24	24

Table S2. Limits of detection (LOD) and limits of quantification (LOQ) of the individual mycotoxins for maize, sorghum, and millet.

Mycotoxin	LOD (μg/kg)			LOQ (μg/kg)		
	Maize	Sorghum	Millet	Maize	Sorghum	Millet
fumonisin B_1	8.2	10	8.2	16.4	20	16.4
fumonisin B_2	11.5	11.3	12.1	23	22.6	24.2
fumonisin B_3	14	14	14	28	28	28
Deoxynivalenol	7	12	10	14	24	20
3-acetyl-deoxynivalenol	10.4	12	10.4	20.8	24	20.8
15-acetyl-deoxynivalenol	5	7	4.4	10	14	8.8
Deoxynivalenol-3-glucoside	15.3	3.77	3.77	30.6	7.54	7.54
Zearalenone	3.25	3.8	3.8	6.5	7.7	7.6
α-zearalenol	4.6	4.6	5.1	9.2	9.2	10.2
β-zearalenol	5	5	7	10	10	14
Zearalenone-14-glucoside	8	7.2	8	16	14.4	16
Nivalenol	35	87.5	81.3	70	175	162.6
Fusarenon-X	20.6	45	73.6	41.2	90	147.2
T-2 toxin	3.6	8	5.4	7.2	16	10.8
HT-2 toxin	6.5	6.5	6.5	13	13	13
Diacetoxyscirpenol	0.32	0.5	0.32	0.64	1	0.64
Neosolaniol	2.2	3	3.7	4.4	6	7.4
